# Chemotherapy for Elderly Ovarian Cancer Patients

**DOI:** 10.4172/2161-0932.1000397

**Published:** 2016-08-31

**Authors:** Sivraj Muralikrishnan, Christos Hatzis, Andrea Katz, Alessandro Santin, Peter E Schwartz, Maysa M Abu-Khalaf

**Affiliations:** 1 Internal Medicine Residency Program, Norwalk Hospital, Norwalk, CT, USA; 2 Section of Medical Oncology, Yale University School of Medicine, Yale Cancer Center, New Haven CT, USA; 3 Cancer Center of South Florida, Palm Beach Gardens, FL, USA; 4 Section of Gynecologic Oncology, Yale University School of Medicine, Yale Cancer Center, New Haven CT, USA

**Keywords:** Ovarian cancer, Elderly, Carboplatin, Paclitaxel, Chemotherapy, Tolerability, Malignancy

## Abstract

**Objective:**

Ovarian cancer is the most lethal cancer involving the female pelvic reproductive system. Its incidence increases with age and with an aging population, its prevalence should also increase. The goal of our retrospective study is to report our experience in treating women over 65 years of age, with a diagnosis of primary ovarian cancer, using standard intravenous chemotherapy.

**Methods:**

The medical records of 78 patients>65 years of age diagnosed with primary ovarian cancer at the Yale Cancer Center between 1996–2006 were retrospectively reviewed and included in our analysis. Patients had stage I–IV disease (stage I n=5, stage II n=8, stage III n=36, stage IV n=25, unknown n=4).

**Results:**

Sixty-three of 78 women (80.8%) completed the prescribed regimen; and 62 women did not require a dose reduction or chemotherapy discontinuation. The most common reason for a dose reduction or treatment discontinuation was fatigue (6.4%), neutropenia (2.6%), patient preference (2.6%), and multiple co-morbidities (2.6%). The most commonly used regimen was paclitaxel 175mg/m^2^ and carboplatin AUC 5. The hazard ratio for PFS and OS for patients who had dose reduction/discontinuation versus those who completed the prescribed dose was 1.3 (95% CI 0.51–3.26) and 0.63 (95% CI 0.17–2.33), respectively.

**Conclusions:**

Our findings illustrate that elderly women are able to tolerate standard chemotherapy with relatively few significant adverse effects. While different treatment modalities in ovarian cancer are continually being evaluated, additional prospective studies are required to better understand the tolerability and efficacy of such treatment in the elderly population.

## Introduction

Cancer is one of the leading causes of morbidity and mortality worldwide, with the number of new cases expected to increase over the next 2 decades [1]. Ovarian cancer is the 6^th^ most commonly diagnosed cancer in women, worldwide. It is the leading cause of death in cancers involving the female pelvic reproductive system in the United States [2]. The incidence of ovarian cancer increases with age, with a median age of diagnosis at 63 years. It is estimated that there will be approximately 21,290 new cases of ovarian cancer in 2015 [3]. With an aging population, the prevalence of ovarian cancer can only be expected to increase. The most common histologic type of ovarian cancer is epithelial and standard treatment, with few exceptions, includes aggressive cytoreductive surgery followed by adjuvant chemotherapy with platinum-based compounds and a taxane. Evidence has shown that elderly patients affected by ovarian cancer have a worse prognosis compared with their younger counterparts, which may be attributed in part to undertreatment [4]. To date, there is mounting evidence that age by itself should not influence whether a patient receives reduced chemotherapy dosing when being treated for an ovarian cancer. The purpose of this retrospective study is to report how women over 65 years of age treated at the Yale Comprehensive Cancer Center, for a diagnosis of primary ovarian cancer, tolerated standard intravenous chemotherapy with carboplatin and paclitaxel, and to report our experience in treating this group of patients.

## Methods and Materials

### Patients

The medical records of 78 patients 65 years of age or older diagnosed with primary ovarian cancer at the Yale Comprehensive Cancer Center between 1996–2006 were retrospectively reviewed and included in our analysis. Women younger than 65 years of age were excluded. Patients were identified through the Tumor Board Registry of Yale-New Haven Hospital. Patients had stage I–IV disease (stage I n=5, stage II n=8, stage III n=36, stage IV n=25, unknown n=4). The diagnosis of a primary ovarian cancer was confirmed by pathology review of biopsy and surgical specimens at our institution. In addition to stage, other demographics and clinical data included grade, histology, chemotherapy dose, number of cycles received, dose reductions, and development of treatment related toxicities. Histological subtypes were collected for 40 of the 78 ovarian cancer cases, and 37 (92.5%) of these cases were of epithelial type. Patients received standard systemic therapy for primary ovarian cancer consisting of carboplatin and paclitaxel. Six out of 78 patients treated earlier in this series also received cyclophosphamide in addition to carboplatin and paclitaxel. The study cohort was retrospectively analyzed and progression-free survival (PFS) was calculated as time from the initial diagnosis to the date of disease recurrence, progression, or last contact. Overall survival (OS) was calculated as time from initial diagnosis to the date of death or date of last documented contact for living patients.

### Pre-treatment evaluation

All patients were evaluated and treated by a gynecologist oncologist. We reviewed patient records and collected the following information: clinical examination, pathology results, diagnostic imaging findings, laboratory results, and treatment plan. Ovarian cancer staging was determined using the 1988 International Federation of Gynecologists and Obstetricians (FIGO) staging system and was evaluated by a combination of histological data and diagnostic imaging. Staging data was available for all but 4 cases.

### Treatment

Patients received intravenous carboplatin at an Area Under the Curve (AUC) dose ranging from 2 to 7.5 and intravenous paclitaxel dose ranging from 40 to 225 mg/m^2^ administered every 3 weeks. Six out of 78 patients also received 600 mg/m^2^ of cyclophosphamide therapy in addition to carboplatin and paclitaxel. Sixty-five out of 78 patients (83.3%) received neoadjuvant chemotherapy. We found that 53 of 78 patients completed 6 cycles or greater of a regimen consisting of at least 175 mg/m^2^ paclitaxel and an AUC ≥ 5 for carboplatin.

### Statistical analysis

We used Cox regression analysis to estimate the hazard ratios associated with relapse and death risks for different disease stages. Kaplan-Meier analysis provided estimates of the survivor functions for the different disease stage groups. Significance was based on the log-rank test. We used the statistical system R (version 3.2.0) for all computations.

## Results

The median age of patients was 76 years (range 65–93). Five patients (6.4%) had stage I disease, 8 patients had stage II (10.3%), 36 patients had stage III (46.2%), and 25 patients had stage IV (32.1%) disease. Four patients (5%) did not have staging data available. The most commonly recorded treatment related toxicity was fatigue (34.6%), followed by nausea (16.7%) and neuropathy (15.4%). Nineteen out of 78 patients (24.4%) did not have any treatment related toxicity recorded (Table 1). The most commonly recorded reason for a dose reduction or treatment discontinuation was fatigue (6.4%) followed by neutropenia (2.6%), patient preference (2.6%), and multiple comorbidities (2.6%). A total of 63 out of 78 women (80.8%) completed the prescribed regimen.

Out of these 63 women, 62 did not have any recorded dose reduction or chemotherapy discontinuation during their treatment course, with only one patient being started on a decreased dose of carboplatin and paclitaxel due to the presence of multiple comorbidities (Table 2). The most commonly used regimen was 175 mg/m^2^ of paclitaxel and AUC 5 Carboplatin (Table 3).

The hazard ratio for PFS and OS for patients who had dose reduction/discontinuation versus those who received the prescribed dose was 1.3 (95% CI 0.51–3.26) and 0.63 (95% CI 0.17–2.33), respectively. Stage IV was associated with significantly greater risk for progression or death, with hazard ratios for PFS and OS between Stage IV versus Stage I/II disease of 3.65 (95% CI 1.37–9.69) and 6.24 (95% CI 1.29–30.2), respectively.

However, women with Stage III disease had similar prognosis with those with Stage I/II disease (hazard ratio for PFS and OS between Stage III versus Stage I/II disease was 1.44 (95% CI 0.59–3.52) and 2.01 (95% CI 0.42–9.56), respectively) (Tables 4 and 5).

**Figure 1:**
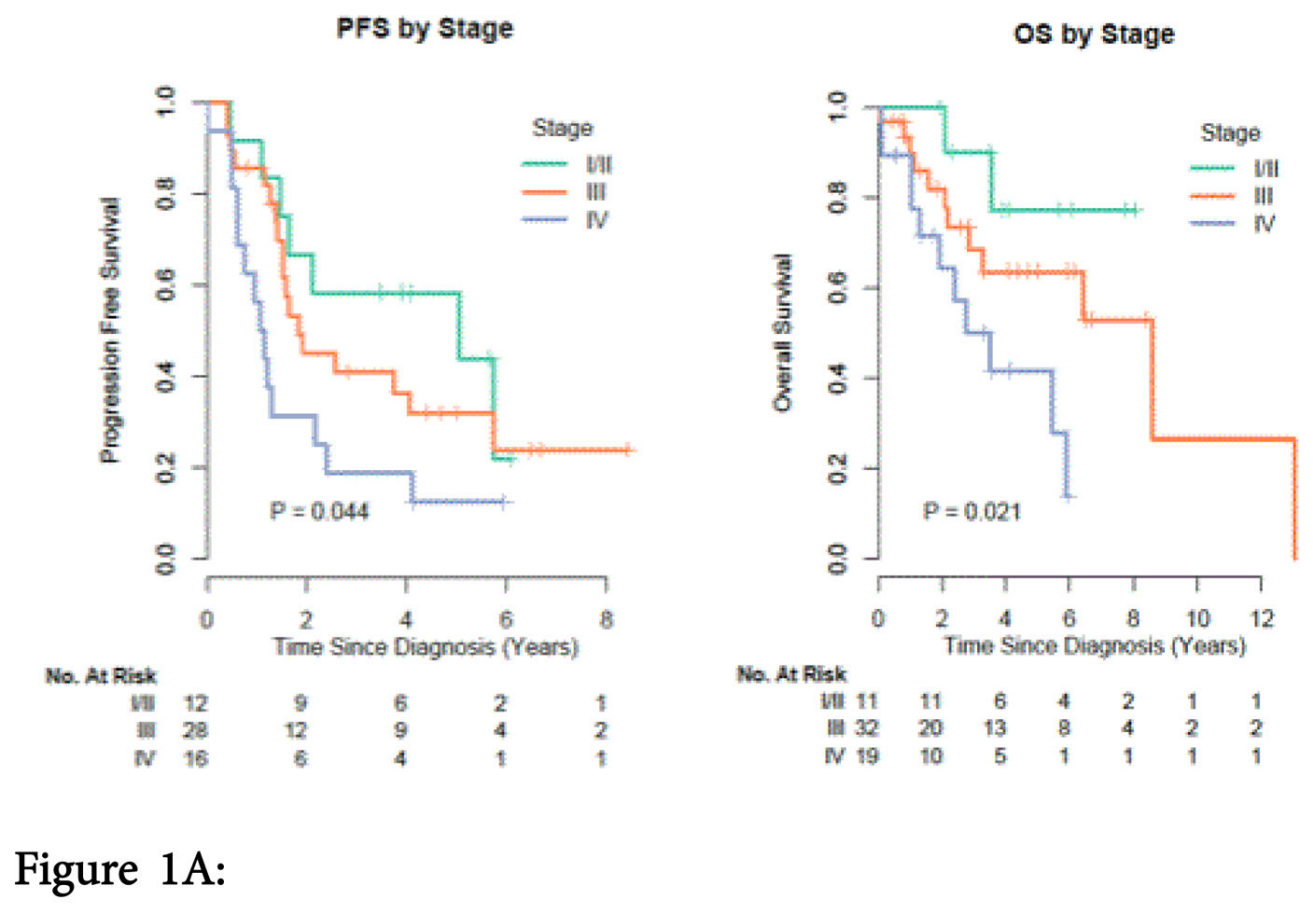

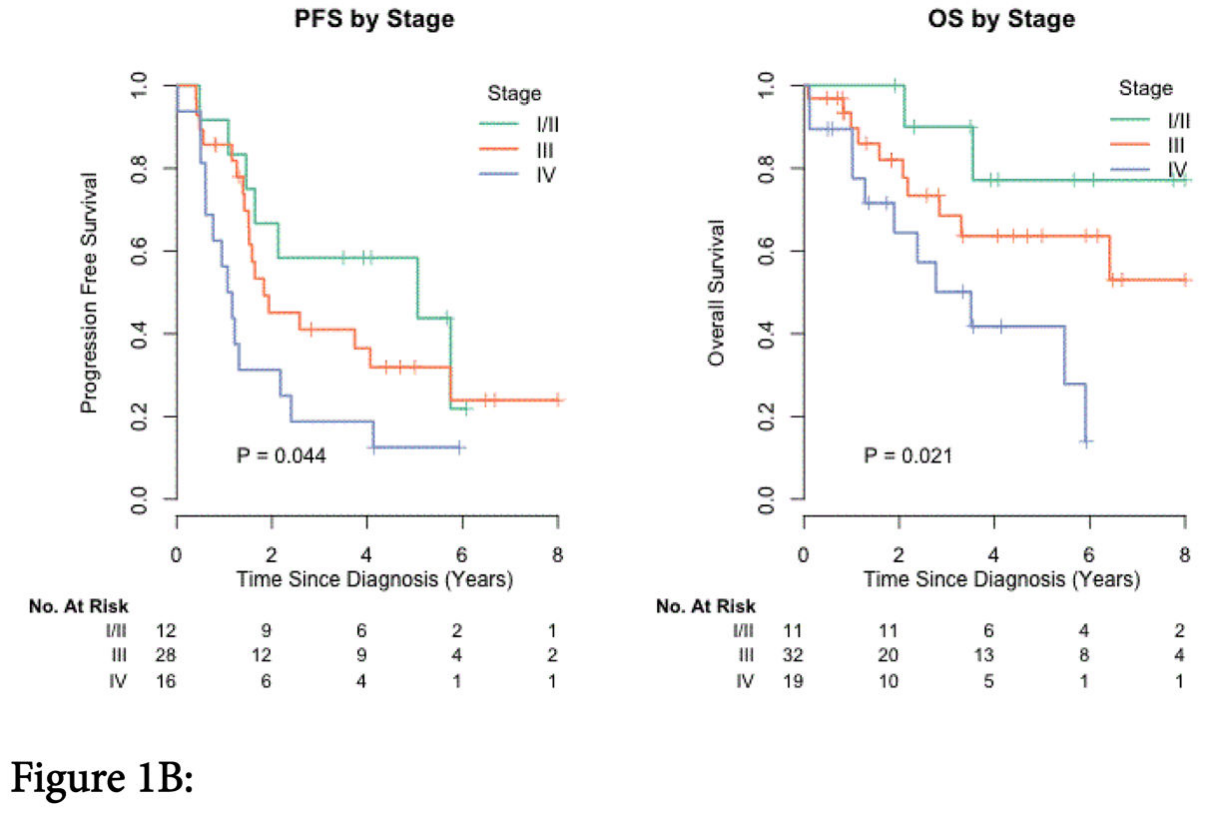
Figure 1A: Kaplan-Meier estimate of progression-free survival by disease stage. P-value is from the log-rank test. Figure 1B: Kaplan-Meier of overall survival by disease stage. P-value is from the log-rank test.

The median PFS for Stage I/II, III, and IV disease was 5.06, 1.83, and 1.12 years, respectively (Figure 1A).

The median OS for stage IV disease was 3.51 years and 8.57 years for stage III disease. The median OS was not reached for stage I/II (Figure 1B).

## Discussion

Retrospective studies have shown that elderly patients with ovarian cancer received reduced chemotherapy doses compared to their younger counterparts as a result of their age. A large population based study evaluating the influence of age on treatment of ovarian cancer found that only 45% of patients aged 70 or older underwent optimal treatment compared to 83% of patients aged younger than 70, even in the absence of co-morbidity [5]. Another study reported that only about half of the women with advanced ovarian cancer over 65 years of age were treated with platinum-based chemotherapy. Those that were treated had an improved survival by 38%, which was similar to the benefits described in other randomized controlled trials involving younger patients [6]. Giuliani et al., reported that in the absence of comorbidity, standard combination chemotherapy was prescribed less often for elderly patients although there was no statistical significance in OS between “young-old” (65–74 years old) and “old-oldest” (>75 years old) [7]. More recently, a study by Fourcadier et al. showed that the probability of a woman over 70 years old receiving standard treatment, in accordance with current recommendations was 50% less than their younger counterparts [8]. In our study, the majority of the elderly patients received standard chemotherapy, which is consistent with our current clinical practice. Our findings help support the idea that age by itself should not influence the decision to treat an elderly patient with optimal chemotherapy.

The majority of patient’s in our study had stage III or IV disease at the time of diagnosis, which is consistent with the findings from the SEER database on ovarian cancer. In the US, only 14.7% of women with ovarian cancer are diagnosed at the local stage (stage I disease) [3]. This may be attributed to the fact that earlier stages can be asymptomatic or present with mild, vague symptoms that do not prompt urgent evaluation. Given the lack of strong and reliable screening tests for ovarian cancer, the US Preventative Services Task Force (USPSTF) currently recommends against screening for ovarian cancer in asymptomatic women [9]. Given the large proportion of patients in whom ovarian cancer is diagnosed at a later stage, including our population, continued research into effective screening tools should be encouraged

The most commonly used regimen in our study was carboplatin (AUC 5) with paclitaxel (175 mg/m^2^), which is considered a standard treatment for ovarian cancer [10–13]. We found that 80.8% of women completed their chemotherapy regimen as prescribed by the treating gynecologist oncologist at the time of initial diagnosis. The limitation of our study is that it is a retrospective analysis, however, our findings lend support to previous studies that addressed the tolerability of combination carboplatin and paclitaxel. In the EWOC2 study, performed by the GINECO group in France between 1998 and 2003, seventy-five women over 70 years of age with ovarian cancer were analyzed for their ability to tolerate intravenous carboplatin (AUC 5) with paclitaxel (175 mg/m^2^) once every 3 weeks for 6 cycles. Results showed that 68.1% of these women were able to complete the above regimen without having dose reduction or stoppage during the mandated 6 cycles [14]. In the study by Villela et al. 75 women with primary ovarian cancer were retrospectively analyzed with regards to their tolerability of standard cytoreductive surgery and chemotherapy treatment based on two different age cohorts. In that study, patients>70 years of age (study group) were compared to those <55 years of age (control group). Overall, the study showed that women in the older age group were able to tolerate aggressive cytoreductive surgery and therapeutic doses of intravenous chemotherapy despite having poorer nutritional status and general health at time of diagnosis [15]. Findings from our study also support the idea that standard chemotherapy treatment is tolerable in the elderly diagnosed with ovarian cancer and should be encouraged in this population in the absence of significant co-morbidity or poor performance status.

With respect to the side effect profile in our study group, we found that fatigue and nausea were most commonly reported. Of those who developed these side effects, only 5 out of the 78 patients required dose reduction or discontinuation of therapy due to fatigue, while there were no patients who had to do so because of nausea. Our findings are consistent with the known side effect profile of these chemotherapeutic agents. Fatigue has been a well-documented adverse effect of chemotherapy in the past [16,17]. In relation to ovarian cancer specifically, a cross-sectional study by Liavaag et al. looking at the somatic and mental morbidities in epithelial ovarian cancer survivors between 1977 and 2003 in a Norwegian Hospital, it was found that chronic fatigue was present in 22% of the patients [18].

Regarding PFS and OS, our study showed that those with Stage IV disease were 3.65 times more likely to suffer from relapse and 6.24 times more likely to die when compared to those with Stage I/II disease regardless of the chemotherapy dosing they received. Furthermore, it should be noted that the median OS for women with Stage I/II was not reached in our study. Our PFS and OS findings in this cohort of elderly ovarian cancer patients are consistent with currently available data that show patients with advanced stages of ovarian cancer have worse prognosis. The reported relative 5-year survival in patients with Stage IV epithelial ovarian cancer is 17% versus 39–59% in Stage III disease and 70–94% in Stage I/II disease [3].

When assessing patients who had dose reduction or discontinuation of treatment versus those who completed their prescribed chemotherapy regimen, there was no statistically significant difference in PFS or OS. However, these findings likely reflect the limited power of our study with a sample size of 78 patients. A study by Trillsch et al., showed that contrary to our findings, there was an improvement in PFS and OS in the elderly treated with optimal versus suboptimal therapy. In the latter study, PFS and OS were compared in elderly patients greater than 70 years of age who received optimal oncologic management (defined as complete tumor resection and platinum-based combination chemotherapy), versus elderly patients who received sub-optimal therapy. Results of the study showed an improved prognosis regarding PFS (18 vs. 11 months) and OS (31 vs. 20 months) when compared to those who received sub-optimal oncologic treatment [19]. Similar to our study, this study also had limited power, as only 47 patients were included in the elderly group (defined as age>70). Future prospective studies, with adequate power, are needed to assess outcome in elderly ovarian cancer patients treated with optimal versus suboptimal treatment. Such studies already exist in other malignancies, such as breast cancer. In a study by Muss et al., 633 women over the age of 65 with early-stage breast cancer were assigned to receive either standard chemotherapy (cyclophosphomide-methotrexate- fluorouracil or cyclophosphomide-doxorubicin) or suboptimal chemotherapy (capecitabine). The women who received suboptimal therapy were more likely to experience a relapsing event or death when compared to optimal therapy [20].

Our retrospective analysis adds to the mounting evidence that elderly women are able to tolerate standard chemotherapy with relatively few significant adverse effects. With the aging population, we can expect to see increasing cases of ovarian cancer worldwide. While different treatment modalities in ovarian cancer are continually being evaluated, additional prospective studies are required to better understand the tolerability and efficacy of such treatment in the elderly population.

## Figures and Tables

**Table 1:** Toxicity.

Toxicity	Number of patients	Percentage of patients
Fatigue	27	34.62
Nausea	13	16.67
Neuropathy	12	15.38
Decreased appetite	10	12.82
Emesis	7	8.97
Constipation	6	7.69
Neutropenia	6	7.69
Alopecia	5	6.41
Diarrhea	5	6.41
Allergy (paclitaxel or carboplatin)	4	5.13
Rash (from paclitaxel)	4	5.13
Anemia	4	5.13
Dehydration	2	2.56
Abdominal pain	2	2.56
Small bowel obstruction	2	2.56
Syncope	2	2.56
Thrombocytopenia	2	2.56
Mucositis	2	2.56
Edema	1	1.28
Bloody bowel movement	1	1.28
Deep vein thrombosis	1	1.28
Cellulitis	1	1.28
Phlebitis	1	1.28
Tachycardia	1	1.28
Gastrointestinal bleeding	1	1.28

**Table 2:** Dose reduction/discontinuation reason.

Dose reduction/ Discontinuation reason	Number of patients	Percentage of patients
Fatigue	5	6.41
Neutropenia	2	2.56
Multiple co-morbidities	2	2.56
Patient preference	2	2.56
Paclitaxel reaction	1	1.28
Neuropathy	1	1.28
Myocardial infarction	1	1.28
Small bowel obstruction	1	1.28
Renal toxicity	1	1.28
Thrombocytopenia	1	1.28
Anemia	1	1.28
Disease progression	1	1.28
Diarrhea	1	1.28

**Table 3:** Chemotherapy régimen.

Regimen	Number of patients	Percentage of patients
P 175 mg/m^2^- C AUC 5	53	67.95
P 175 mg/m^2^ - C AUC 6	4	5.13
P 175 mg/m^2^ - C AUC 7.5	3	3.85
P 175 mg/m^2^ - C AUC 5 - Cyt 600 mg/m^2^	2	2.56
P 135 mg/m^2^ - C AUC 5	2	2.56
P 135 mg/m^2^ - C AUC 3	2	2.56
P 175 mg/m^2^ - C AUC 6.5	1	1.28
P 175 mg/m^2^ - C AUC 4	1	1.28
P 120 mg/m^2^ - C AUC 4	1	1.28
P 80 mg/m^2^ - C AUC 5 - Cyt 600 mg/m^2^	1	1.28
P 80 mg/m^2^ - C AUC 5	1	1.28
P 60 mg/m^2^- C AUC 6 - Cyt 600 mg/m^2^	1	1.28
P 60 mg/m^2^ - C AUC 5 - Cyt 600 mg/m^2^	1	1.28
P 60 mg/m^2^ - C AUC 6 to 5	1	1.28
P 40 mg/m^2^ - C AUC 6 to 5	1	1.28
P 225 to 175 mg/m^2^ - C AUC 6 to 5	1	1.28
P 175 to 135 mg/m^2^ - C AUC 5 to 4	1	1.28
P 135 to 80 mg/m^2^ - C AUC 5 - Cyt 600 mg/m^2^	1	1.28

P=Paclitaxel, C=Cyclophosphamide, Cyt=Cytoxan, AUC=Area under Curve

**Table 4:** Progression free survival.

Variable	Hazard ratio	95% Confidence Interval	p-value
Stage III vs I/II	1.44	0.59–3.52	0.42
Stage IV vs I/II	3.65	1.37–9.69	0.0095
Dose reduction/discontinuation vs Prescribed dose	1.3	0.51–3.26	0.58
Age at diagnosis>76 vs ≤ 76	0.54	0.26–1.11	0.095

**Table 5:** Overall Survival.

Variable	Hazard Ratio	95% Confidence Interval	p-value
Stage III vs I/II	2.01	0.42–9.56	0.38
Stage IV vs I/II	6.24	1.29–30.2	0.023
Dose reduction/ discontinuation vs Prescribed dose	0.63	0.17–2.33	0.49
Age at Dx>76 vs ≤ 76	0.72	0.30–1.74	0.47
